# *Elbamycellarosea* gen. et sp. nov. (Juncigenaceae, Torpedosporales) isolated from the Mediterranean Sea

**DOI:** 10.3897/mycokeys.55.35522

**Published:** 2019-06-19

**Authors:** Anna Poli, Elena Bovio, Gerard Verkley, Valeria Prigione, Giovanna Cristina Varese

**Affiliations:** 1 Department of Life Sciences and Systems Biology, Mycotheca Universitatis Taurinensis (MUT), University of Torino, Viale Mattioli 25, 10125 Torino, Italy University of Torino Torino Italy; 2 Westerdijk Fungal Biodiversity Institute, Uppsalalaan 8, 3584CT Utrecht, The Netherlands Westerdijk Fungal Biodiversity Institute Utrecht Netherlands

**Keywords:** Marine fungi, new taxon, TBM clade

## Abstract

*Elbamycellarosea***sp. nov.**, introduced in the new genus *Elbamycella*, was collected in the Mediterranean Sea in association with the seagrass *Posidoniaoceanica* and with the brown alga *Padinapavonica*. The affiliation of the new taxon to the family Juncigenaceae is supported by both morphology and phylogenetic inference based on a combined nrSSU and nrLSU sequence dataset. Maximum-likelihood and Bayesian phylogeny proved *Elbamycella***gen. nov.** as a distinct genus within Juncigenaceae. The new genus has been compared with closely related genera and is characterised by a unique suite of characters, such as ascospores with polar appendages and peculiar shape and dimension of ascomata and asci.

## Introduction

Marine fungi are a considerable part of the huge diversity of microorganisms that inhabit the Oceans (Richards et al. 2012). These organisms, which are distributed worldwide, live on a broad range of biotic and abiotic substrates (e.g. algae, sponges, corals, sediments) (Jones and Pang 2012) and are divided in two major ecological categories, namely obligate and facultative marine fungi. The former grow and reproduce exclusively in the sea, the latter are terrestrial species that can actively grow and reproduce in marine environments. Those fungi whose obligate or facultative marine nature is undefined are called marine-derived (Raghukumar 2017).

The number of marine fungi has been estimated to exceed 10,000 taxa, but the most recent update in marine mycology listed only 1,206 species belonging to Ascomycota, Basidiomycota, Chytridiomycota, and Mucoromycota. Thus fungal diversity is largely undescribed (Pang and Jones 2017).

In an attempt to clarify the phylogeny of the genera *Swampomyces* Kohlm. & Volkm.-Kohlm. and *Torpedospora* Meyers, Sakayaroj et al. (2005) recognised a distinct lineage of marine Ascomycota within the class Sordariomycetes that was then named TBM (*Torpedospora*/*Bertia*/*Melanospora*) clade (Schoch et al. 2007). Following a re-evaluation of the marine fungi affiliated to the TBM clade, together with the terrestrial genus *Falcocladium*, new families were introduced to accommodate its four subclades: Juncigenaceae, Etheirophoraceae, Falcocladiaceae, and Torpedosporaceae, all belonging to the order Torpedosporales (Jones et al. 2014; Abdel-Wahab et al. 2018). Based on phylogeny and morphological data, Maharachchikumbura et al. (2015) introduced the order Falcocladiales (Falcocladiaceae) under the class Sordariomycetes.

Recently, during a survey focused on the fungal diversity in the Mediterranean Sea, two unidentified Sordariomycetes were isolated from the seagrass *Posidoniaoceanica* (L.) Delile (Panno et al. 2013) and from the brown alga *Padinapavonica* (L.) Thivy (Garzoli et al. 2018). The present paper provides a phylogenetic and morphological study of the two strains that turn out to represent a new genus within the family Juncigenaceae.

## Material and methods

### Fungal isolates

The fungal isolates investigated in this paper were previously retrieved from *P.oceanica* (MUT 4937 = CBS 130520) and *P.pavonica* (MUT 5443) from the coastal waters of Elba island, in the Mediterranean Sea (Panno et al. 2013; Garzoli et al. 2018) (Table 1). The two strains were originally isolated on corn meal agar medium supplemented with sea salts (CMASS; 3.4% w/v sea salt mix, Sigma-Aldrich, Saint Louis, USA, in ddH_2_O) and are preserved at the *Mycotheca Universitatis Taurinensis* (MUT), Italy, and CBS Collection of the Westerdijk Fungal Biodiversity Institute, the Netherlands.

**Table 1.** Dataset used for phylogenetic analysis. Genbank sequences including newly generated ITS, LSU and SSU amplicons relative to *Elbamycellarosea* sp. nov. and *Torpedosporaambispinosa*MUT 3537.

Species	Strain Code	Source	ITS	SSU	LSU
** HYPOCREALES **					
*Bionectriapityrodes* (Mont.) Schroers	GJS95-26	Bark	–	AY489696	AY489728
*Clonostachysrosea* (Link.) Schroers	GJS90-227	Bark	–	AY489684	AY489716
*Cordycepsmilitaris* (L.) Fr.	NRRL 28021	–	–	AF049146	AF327374
*Fusariumsolani* (Mart.) Sacc.	GJS89-70	Bark	–	AY489697	AY489729
*Trichodermadeliquescens* (Sopp.) Jaklitsch	ATCC 208838	Pine wood	–	AF543768	AF543791
** MICROASCALES **					
*Cephalotrichumstemonitis* (Pers.) Nees	AFTOL 1380	Seed	–	DQ836901	DQ836907
*Halosphaeriaappendiculata* Linder	NTOU4004	Driftwood	–	KX686781	KX686782
*Lignincolalaevis* Hohnk	JK5180A	Wooden stake	–	U46873	U46890
*Microascustrigonosporus* Emmons & Dodge	AFTOL 914	–	–	DQ471006	DQ470958
*Nimbosporaeffusa* Kock	NTOU4018	Intertidal wood	–	KX686793	KX686794
*Noheaumiumi* Kohlm. & Volkm. Kohlm.	NTOU4006	Driftwood	–	KX686795	KX686796
*Petriellasetifera* (Schmidt) Curzi	AFTOL 956	Wood panel in coastal water	–	DQ471020	DQ470969
** TORPEDOSPORALES **					
** Etheirophoraceae **					
*Etheirophorablepharospora* (Kohlm. & E. Kohlm.) Kohlm. & Volkm. Kohlm.	JK5397A	Bark on submerged proproots	–	EF027717	EF027723
*E.unijubata* Kohlm. & Volkm. Kohlm.	JK5443B	Submerged wood	–	EF027718	EF027725
*Swampomycesarmeniacus* Kohlm. & Volkm. Kohlm.	JK5059C	Mangroves	–	EF027721	EF027728
*S.triseptatus* Hyde & Nakagiri	CY2802	Submerged wood	–	AY858942	AY858953
** Juncigenaceae **					
*Juncigenaadarca* Kohlm., Volkm. Kohlm. & Erikss	JK5548A	* Juncus roemerianus *	–	EF027720	EF027727
*J.fruticosae* (Abdel-Wahab, Abdel-Aziz & Nagah.) Mill. & Shearer	EF14	Driftwood	–	GU252146	GU252145
	IMI391650	Driftwood	–	NG_061097	NG_060791
*Khaleijomycesmarinus* Abdel-Wahab	MD1348	Driftwood	–	MG717679	MG717678
*Marinokulatichaetosa* (Kohlm.) Jones & Panf	BCRC FU30271	Driftwood	–	KJ866929	KJ866931
	BCRC FU30272	Driftwood	–	KJ866930	KJ866932
*Fulvocentrumaegyptiacum* (Abdel-Wahab, El-Shar. & Jones) Jones & Abdel-Wahab	CY2973	Mangroves	–	AY858943	AY858950
*F.clavatisporum* (Abdel-Wahab, El-Shar. & Jones) Jones & Abdel-Wahab	LP83	Mangroves	–	AY858945	AY858952
***Elbamycellarosea* sp. nov.**	MUT 4937	* P. oceanica *	MK775496*	MK775501*	MK775499*
***Elbamycellarosea* sp. nov.**	MUT 5443	* P. pavonica *	MK775497*	MK775502*	MK775500*
** Torpedosporaceae **					
*Torpedosporaambispinosa* Kohlm.	CY3386	Driftwood	–	AY858941	AY858946
	BCC16003	Driftwood	–	AY858940	AY858949
	MUT 3537	Driftwood	MK775503*	MK775498*	MK775495*
*T.mangrovei* (Abdel-Wahab & Nagah.) Jones & Abdel-Wahab	NBRC 105264	Mangroves	NR_138418	GU252150	GU252149
*T.radiata* Meyers	BCC11269	Driftwood	–	AY858938	AY858948
	PP7763	Driftwood	–	AY858939	AY858947
** FALCOCLADIALES **					
** Falcocladiaceae **					
*Falcocladiummultivesiculatum* Silveira, Alfenas, Crous & Wingf	CBS 120386	Leaves	–	JF831928	JF831932
*F.sphaeropedunculatum* Crous & Alfenas	CBS 111292	Leaves	–	JF831929	JF831933
*F.thailandicum* Crous & Himaman	CBS 121717	Leaves	–	JF831930	JF831934
*F.turbinatum* Somrith., Sudhom, Tippawan & Jones	BCC22055	Dead leaves	–	JF831931	JF831935
** XYLARIALES **					
*Daldiniaconcentrica* (Bolton) Ces. & De Not.	ATCC 36659	*Fraxinus* sp.	–	U32402	U47828
*Hypoxylonfragiforme* (Pers.) Kickx	HKUCC 1022	Bark	–	AY083810	AY083829
*Xylariahypoxylon* (L.) Grev.	AFTOL 51	Rotting wood	–	AY544692	AY544648

* = newly generated sequences

### Morphological analysis

MUT 4937 and MUT 5443 were pre-grown on CMA-sea water (CMASW; 17 g corn meal agar in 1 L of sea water) for one month at 21 °C prior to inoculation in triplicate onto Petri dishes (9 cm Ø) containing CMASS, CMASW, Potato Dextrose Agar (PDA) SS or PDASW. Petri dishes were incubated at 10 °C and 21 °C. The colony growth, together with macroscopic and microscopic traits, were monitored for 28 days.

Reproductive structures were observed and captured using an optical microscope (Leica DM4500B, Leica microsystems GmbH, Germany) equipped with a camera (Leica DFC320, Leica microsystems GmbH, Germany). Macro- and microscopic features were compared with the available description of Juncigenaceae (Kohlmeyer et al. 1997; Abdel-Wahab et al. 2001; Jones et al. 2014; Abdel-Wahab et al. 2018).

### DNA extraction, PCR amplification, and data assembling

Genomic DNA was extracted from about 100 mg of mycelium carefully scraped from CMASS plates. Mycelium was transferred to a 2 mL Eppendorf tubes and disrupted in a MM400 tissue lyzer (Retsch GmbH, Haan, Germany). Extraction was accomplished using a NucleoSpin kit (Macherey Nagel GmbH, Duren, DE, USA) following the manufacturer’s instructions. The quality and quantity of DNA samples were measured spectrophotometrically with Infinite 200 PRO NanoQuant (TECAN, Switzerland) and stored at −20 °C.

The primer pairs ITS1/ITS4 (White et al. 1990), LROR/LR7 (Vilgalys and Hester 1990), and NS1/NS4 (White et al. 1990) were used to amplify the partial sequences of the internal transcribed spacers including the 5.8S rDNA gene (ITS), partial large ribosomal subunit (nrLSU), and partial small ribosomal subunit (nrSSU), respectively. Ribosomal genes were amplified in a T100 Thermal Cycler (Bio-Rad, Hercules, CA, USA), as previously described (Bovio et al. 2018). Reaction mixtures consisted of 60–80 ng DNA template, 10× PCR Buffer (15 mM MgCl_2_,500 mM KCl, 100 mM Tris-HCl, pH 8.3), 200 µM each dNTP, 1 μM each primer, 2.5 U Taq DNA Polymerase (Qiagen, Chatsworth, CA, USA), in 50 μL final volume. Following visualization of the amplicons on a 1.5% agarose gel stained with 5 mL 100 mL^−1^ ethidium bromide, PCR products were purified and sequenced at Macrogen Europe Laboratory (Madrid, Spain). The resulting ABI chromatograms were processed and assembled to obtain consensus sequences using Sequencer v. 5.0 (GeneCodes Corporation, Ann Arbor, Michigan, USA http://www.genecodes.com). Newly generated sequences were deposited in GenBank (Table 1).

### Sequence alignment and phylogenetic analysis

A dataset consisting of nrLSU and nrSSU was assembled on the basis of BLASTn results and of a recent phylogenetic study focused on Torpedosporales (Abdel-Wahab et al. 2018). Reference sequences were retrieved from GenBank. Although nrITS regions were amplified for MUT 4937 and MUT 5443, they were not used for phylogenetic analyses, due to the lack of available ITS sequences for the strains present in the tree. Alignments were generated using MUSCLE (default conditions for gap openings and gap extension penalties), implemented in MEGA v. 7.0 (Molecular Evolutionary Genetics Analysis), visually inspected and trimmed by TrimAl v. 1.2 (http://trimal.cgenomics.org) to delimit and discard ambiguously aligned regions. nrITS alignment was not performed, due to the lack of reference sequences. Preliminary analyses suggested no incongruence among single-loci phylogenetic trees, as assessed through the Incongruence Length Difference (ILD) test (de Vienne et al. 2007). As a consequence, alignments were concatenated into a single data matrix with SequenceMatrix (Vaidya et al. 2011). The appropriate evolutionary model under the Akaike Information Criterion (AIC) was determined with jModelTest 2 (Darriba et al. 2012).

**Figure 1.**
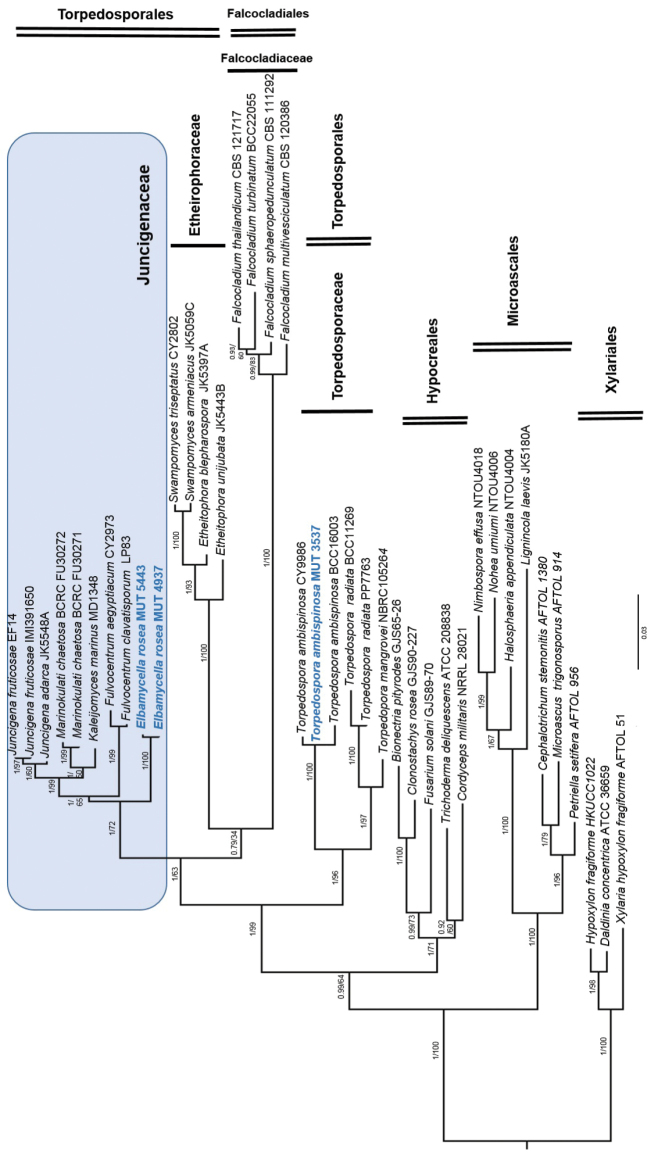
Phylogenetic inference of *Elbamycellarosea* sp. nov. based on a combined nrSSU and nrLSU dataset. The tree is rooted to *Xylariahypoxylon*. Branch numbers indicate BPP/MLB values; Bar = expected changes per site (0.03).

Phylogenetic inference was estimated using both Maximum Likehood (ML) and Bayesian Inference (BI). The ML analysis was performed using RAxML v. 8.1.2 (Stamatakis 2014) under GTR + I + G evolutionary model (best model) and 1,000 bootstrap replicates. Support values from bootstrapping runs (MLB) were mapped on the globally best tree using the “-f a” option of RAxML and “-x 12345” as a random seed to invoke the novel rapid bootstrapping algorithm. BI was performed with MrBayes 3.2.2 (Ronquist et al. 2012) with the same substitution model (GTR + I + G). The alignment was run for 10 million generations with two independent runs each containing four Markov Chains Monte Carlo (MCMC) and sampling every 100 iterations. The first quarter of the trees were discarded as “burn-in”. A consensus tree was generated using the “sumt” function of MrBayes and Bayesian posterior probabilities (BPP) were calculated. Consensus trees were visualized in FigTree v. 1.4.2 (http://tree.bio.ed.ac.uk/software/figtree). Members of Xylariales (i.e. *Xylariahypoxylon*, *Hypoxylonfragiforme*, and *Daldiniaconcentrica*) were used as outgroup taxa. Due to topological similarity of the two resulting trees, only ML analysis with MLB and BPP values is reported (Fig. 1).

Sequence alignments and phylogenetic tree were deposited in TreeBASE (http://www.treebase.org, submission number 24426).

## Results

### Phylogenetic inference

Preliminary analyses were carried out individually with nrSSU and nrLSU. The topology of the single-locus trees was very similar and the ILD test confirmed the congruence between them (*p* = 0.001). The combined dataset consisted of an equal number of nrSSU and nrLSU sequences relative to 39 taxa (including MUT 4937 and MUT 5443) that represented 23 genera and 33 species (Table 1). Nine sequences (3 nrSSU, 3 nrLSU, and 3 nrITS) were newly generated while 72 were retrieved from GeneBank. SSU and LSU sequences relative to MUT 4937 and MUT 5443 displayed 100% and 99% similarity (3 bp substitutions). The combined dataset had an aligned length of 1676 characters, of which 1208 were constant, 92 were parsimony-uninformative and 376 parsimony informative (TL = 315, CI = 0.603715, RI = 0.802773, RC = 0.549296, HI = 0.396285).

The two isolates MUT 4937 and MUT 5443 clustered within the family Juncigenaceae together with *Marinokulatichaetosa*, *Khaleijomycesmarinus*, *Juncigenaadarca*, *J.fruticosae*, *Fulvocentrumaegyptiacum*, and *F.clavatisporum* (Fig. 1; BPP = 1; MLB = 72%) and formed a strongly supported monophyletic lineage (Fig. 1; BPP = 1; MLB = 100%) indicating that these strains are phylogenetically different from the other members of the family.

### Taxonomy

#### Elbamycella

Taxon classificationFungiTorpedosporalesJuncigenaceae

gen. nov. A. Poli, E. Bovio, V. Prigione & G.C. Varese

Mycobank: MB830648

##### Type species.

*Elbamycellarosea* sp. nov.

##### Etymology.

In reference to the geographic isolation site, Elba Island, Tuscany (Italy)

##### Phylogenetic placement.

Juncigenaceae, Sordariomycetes, Ascomycota. The genus *Elbamycella* gen. nov. clusters together with genera *Marinokulati*, *Khaleijomyces*, *Juncigena*, and *Fulvocentrum* (Fig. 1).

##### Description.

Ascomata superficial, erumpent or immersed, perithecial, scattered or gregarious, olivaceous-brown to black at maturity, globose, subglobose, ovoid or pyriform, glabrous; ostiolar neck long, pale-coloured; peridium of textura prismatica in the outer layers and textura globulosa in the inner layers. Asci evanescent, hyaline, cylindrical to clavate.

Ascopores cylindrical rounded at both ends, thin-walled, hyaline, straight or slightly curved, 3-septate, bearing subpolar, appendages.

Asexual morph unknown.

#### Elbamycella rosea

Taxon classificationFungiTorpedosporalesJuncigenaceae

sp. nov. A. Poli, E. Bovio, V. Prigione & G.C. Varese

Mycobank: MB830649

Figures 2 , 3

##### Type.

Italy, Tuscany, Mediterranean Sea, Elba Island (LI), Ghiaie ISL, 14–15m depth, 42°49’04”N, 10°19’20”E, on the brown alga *Padinapavonica*, 20 March 2010, R. Mussat-Sartor and N. Nurra, MUT 5443 holotype, living culture permanently preserved in metabolically inactively state by deep-freezing at *Mycotheca Universitatis Taurinensis*. A dried specimen of this culture grown on CMASS and CMASW has been deposited in the herbarium of the Department of Life Sciences and Systems Biology (TO Cryptogamia 3446).

##### Additional material examined.

Italy, Tuscany, Mediterranean Sea, Elba island (LI), Ghiaie ISL, 14–15m depth, 42°49’04”N, 10°19’20”E, on the seagrass *Posidoniaoceanica*, 20 March 2010, R. Mussat-Sartor and N. Nurra, MUT 4937 = CBS 130520.

##### Etymology.

In reference to the colour of the colony on the culture media.

##### Description.

**Figure 2.**
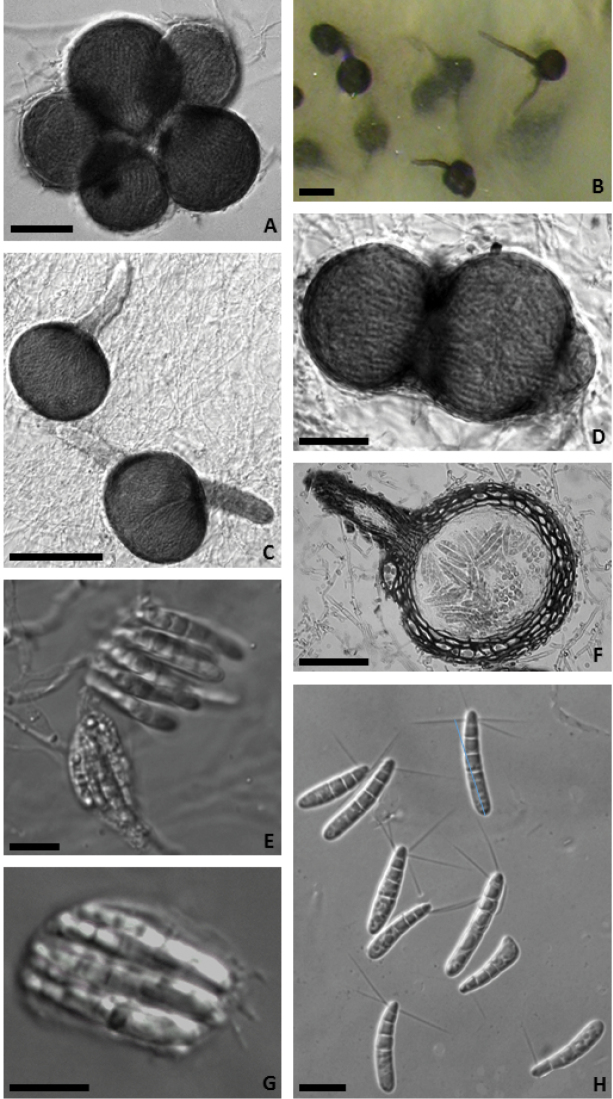
*Elbamycellarosea* sp. nov. **A, D** group of young subglobose ascomata **B, C** globose ascomata with one or two necks **E** immature (bottom) and dehiscent (top) asci **F** ascoma in cross section **G** mature ascus with 8 ascospores **H** ascospores. Scale bars: 50 µm (**A, D, F**); 100 µm (**B, C**); 10 µm (**E, G, H**).

Ascomata were produced on both CMASS and CMASW at 21 °C only, after 28 days of incubation. Mycelium hyaline to pale brown consisting of smooth-walled hyphae 2.5–4 µm wide (Fig. 2A–D, F).

Ascomata perithecial, scattered or gregarious (from 2 to 6-8), superficial, erumpent or immersed, olivaceous-brown to black at maturity, globose, subglobose, ovoid or pyriform, glabrous, up to 100–140 µm diameter; ostiolar neck, pale-coloured, single (sometimes 2, rarely 3), 55–70 µm long and 20–50 µm wide at the base; peridium 5–10 µm thick of textura prismatica in the outer layers and textura globulosa in the inner layers with cells with olivaceous-brown walls, in the neck consisting of hyaline, more elongated cells, from which numerous hyaline blunt hyphal projections 5–15 × 3–5 µm arise. Asci evanescent, hyaline, cylindrical to clavate 22–26 × 12–16 µm containing 8 spores; sterile elements not observed (Fig. 2E, G).

Ascopores cylindrical 23–28 × 4–5 µm, rounded at both ends, thin-walled, hyaline, straight or slightly curved, 3-septate, with a large basal cell 10–15 µm long and 3 shorter, upper cells, slightly constricted around the septa, the apical cell somewhat attenuated just below the blunt tip, bearing 3(4) subpolar, straight or slightly bent, acuminate, hyaline, smooth-walled cellular appendages 10–20 µm long (about 0.5–1 µm wide). In some spores the apical cell is divided by an additional septum; each cell of the spore contains a few oil-droplets 1.5–3.0 µm diameter (Fig. 2H).

Asexual morph not observed.

##### Colony description.

**Figure 3.**
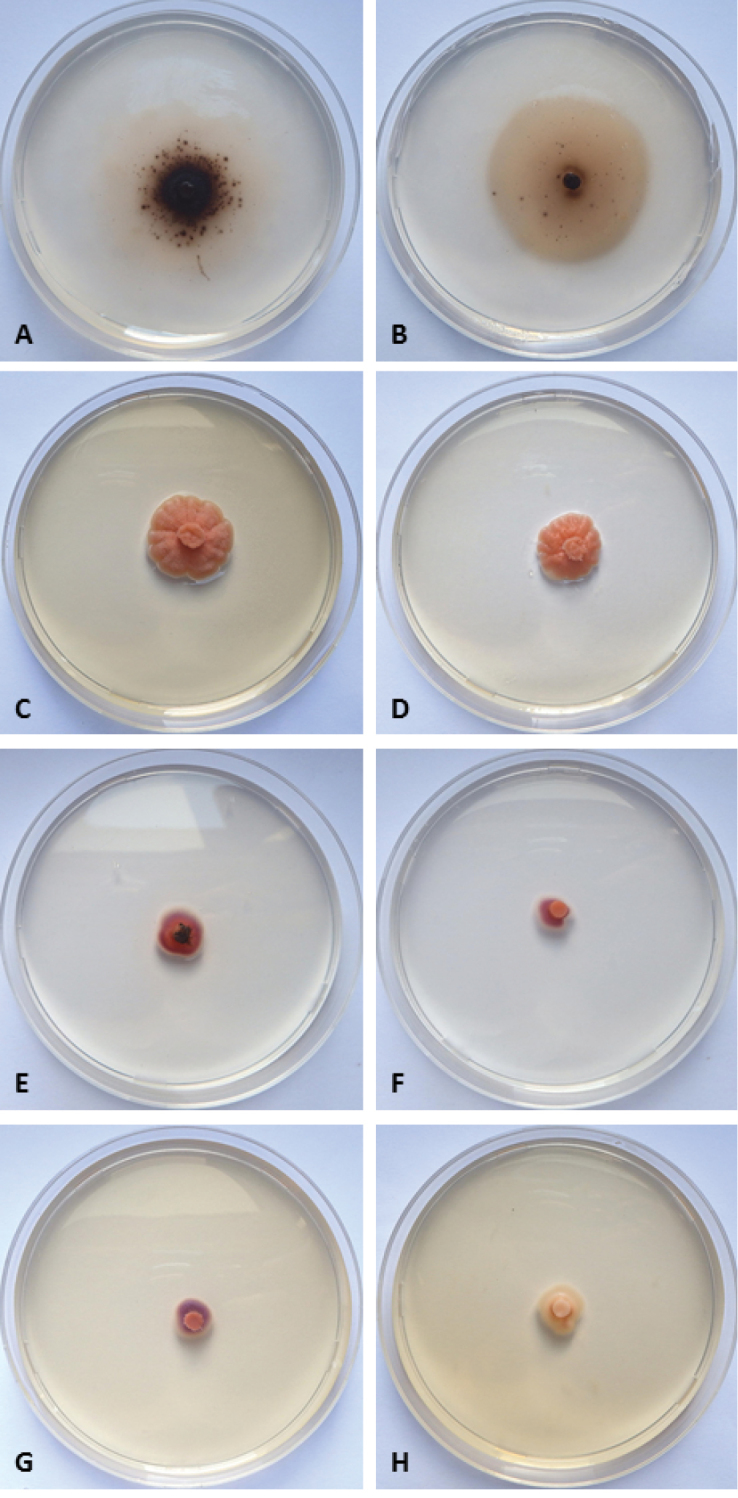
*Elbamycellarosea* sp. nov.: 28-days-old colonies at 21 °C on **A**CMASW**B**CMASS**C** PDASW **D** PDASS; 28-days-old colonies at 10 °C on **E**CMASW**F**CMASS**G** PDASW **H** PDASS.

Colonies reaching 21–23 mm diameter on CMASW and 19–29 mm diameter on CMASS in 28 days at 21 °C, plane, thin, mycelium mainly submerged. Colonies pale pink in the centre becoming brown with age, colourless at the margins. Black spots due to ascomata groups in fruiting colonies. Reverse of the same colour of the surface (Fig. 3A, B).

Colonies on PDASW and PDASS reaching 10–14 mm diameter in 28 days at 21 °C, convolute, developing in height with irregular margins, salmon. Reverse of the same colour of the surface (Fig. 3C, D).

At 10 °C colony growth on all media very poor, attaining 5–8 mm diameter in 28 days. Colonies plane to slightly convolute with regular margins, pale pink to cyclamen. Reverse of the same colour of the surface (Fig. 3E–H).

**Table 2.** Comparison of the main sexual morpholgical features of genera belonging to Juncigenaceae.

**Fungus**	**Ascomata**	**Periphyses/ Paraphyses**	**Asci**	**Ascospores**	**Reference**
* Marinokulati chaetosa *	Immersed to superficial, dark brown, ostiolate, papillate; neck 20 × 70 µm	Both present, septate, wide	102−135 × 12−18 µm; cylindrical to clavate, attenuate at the base, thick-walled at the apex, containing 8 spores	25.5−36.5 × 7.5−11.5 µm; 3-septate, hyaline, fusiform to ellipsoidal with polar and equatorial appendages	Jones et al. 2014
* Khaleijomyces marinus *	Superficial to immersed, hyaline to yellow-orange to reddish brown, ostiolate; 110−175 × 100−115 µm; neck 120-175 × 40-50 µm	Periphyses present in the neck	60−98 × 12−16 µm; cymbiform to fusiform, thin-walled, with no apical apparatus, containing 8 spores	12−26 × 6-8 µm; 1-4-septate; ellipsoidal to fusiform; hyaline, smooth-walled	Abdel-Wahab et al. 2018
* Juncigena adarca *	Immersed, ostiolate, papillate, 225−400 × 135-200 µm; neck 85−170 × 50−85 µm	Both present; Paraphyses thin, branched, septate	115−140 × 10−13 µm; fusiform to cylindrical, short pedunculate, apical apparatus with a ring, containing 8 spores	26.5−34.5 × 6−7 µm; 3-septate, hyaline, fusiform to ellipsoidal, no appendages, smooth wall, constricted	Kohlmeyer et al. 1997
* Fulvocentrum aegyptiacum *	Immersed, dark brown, ostiolate; 240−280 × 170−190 µm; neck 70−80 µm diameter	Both present; Paraphyses numerous, in a gel, unbranched	145−155 × 9−10 µm; Short pedicellate, apically thickened, containing 8 spores	15−20 × 6−8 µm; 3-septate, ellipsoidal, hyaline	Abdel-Wahab et al. 2001
* Fulvocentrum clavatisporum *	Immersed, dark brown, ostiolate; 160−170 × 160−190 µm; neck 50 µm long	Both present; Paraphyses numerous, in a gel, unbranched	80−96 × 10−13µm; Pedicellate, apically thickened, containing 8 spores	25−28 × 5−6 µm; 3-septate, clavate, hyaline	Abdel-Wahab et al. 2001
* Fulvocentrum rubrum *	Erumpent to superficial, olive-brown to dark brown, ostiolate; 145−270 µm; neck 310−390 × 50−55 µm	Both present	Fusiform or obclavate, 95−130 × 13−19 µm; persistent, thin-walled, containing 8 spores	Ellipsoidal to clavate, no appendages, 25−33 × 6−9 µm; hyaline to faint apricot, smooth walled, 3−5-septate	Abdel-Wahab et al. 2019
*Elbamycellarosea* sp. nov.	Superficial, erumpent or immersed; 100–140 µm diam; olivaceous-brown to black; ostiolar neck 55−70 × 20–50 µm		Cylindrical to clavate 22−26 × 12−16 µm containing 8 spores	Cylindrical 23−28 × 4−5 µm; hyaline, generally 3-septate, bearing 3(4) subpolar appendages 10–20 × 0.5–1 µm	This study

## Discussion

The novel genus *Elbamycella* is introduced in this study and has been compared to the closest genera. Herein, the two strains MUT 4937 and MUT 5443 represented a new species that formed a well-supported cluster phylogenetically distant from the related genera of Juncigenaceae.

From a morphological point of view, the relatedness with the other species belonging to Juncigenaceae is confirmed by i) 3-septate spores (1–4 only in *K.marinus*), ii) 8-spored asci, and iii) ascomata with an elongated neck (Kohlmeyer et al. 1997; Abdel-Wahab et al. 2001; Abdel-Wahab et al. 2010; Jones et al. 2014; Abdel-Wahab et al. 2018). *Elbamycellarosea* sp. nov. is furthermore characterised by the presence of polar appendages on the ascospores. *Marinokulatichaetosa* displays this feature too, although it can be distinguished from *E.rosea* sp. nov. by additional, equatorially placed appendages. Additonally, in the new species, spores are cylindrical, not fusiform-ellipsoidal as in *M.chaetosa* (Jones et al. 2014). *Khaleijomycesmarinus*, *Juncigenaadarca*, *Fulvocentrumaegyptiacum*, *F.clavatisporum*, and the recently described *F.rubrum* differ in the shape and dimensions of the ascospores (Jones et al. 2014; Abdel-Wahab et al. 2018; Abdel-Wahab et al. 2019); generally asci and ascomata are larger than those observed in *E.rosea* sp. nov.

As no sexual form is known for *J.fruticosae*, the comparison with *E.rosea* sp. nov. is not possible. However, the similarity or identity to this species is excluded by the phylogenetical distance.

Ecologically, the described Juncigenaceae are species having a marine origin. So far, they have all been retrieved from driftwood in the intertidal of salt marshes (Kohlmeyer et al. 1997; Jones et al. 2014). The new species was found for the first time underwater, in association with the seagrass *P.oceanica* and the brown alga *P.pavonica*, two different organisms that were sampled in close proximity. This could be related to a successful spore dispersal; indeed polar appendages are known to facilitate floatation and attachment (Overy et al. 2019).

## Supplementary Material

XML Treatment for Elbamycella

XML Treatment for Elbamycella rosea
